# Cardiovascular Involvement in Systemic Lupus Erythematosus: Focus on Arrhythmias

**DOI:** 10.3390/diagnostics16030372

**Published:** 2026-01-23

**Authors:** Monica Claudia Dobos, Veronica Ungurean, Diana Elena Costan, Mara Russu, Anca Ouatu, Paula Cristina Morariu, Alexandru Florinel Oancea, Maria Mihaela Godun, Diana-Elena Floria, Dragos Traian Marcu, Genoveva Livia Baroi, Silviu Marcel Stanciu, Anton Knieling, Daniela Maria Tanase, Codrina Ancuta, Mariana Floria

**Affiliations:** 1 Department of Medical Sciences II, “Grigore T. Popa” University of Medicine and Pharmacy, 700115 Iasi, Romania; monicabalac329@yahoo.com (M.C.D.); ungurean.veronica@yahoo.com (V.U.); costan.diana0198@yahoo.com (D.E.C.); mara.russu@umfiasi.ro (M.R.); 2 Department of Rheumatology, Clinic Rehabilitation Hospital, 700661 Iasi, Romania; 3 Department of Medical Sciences I, “Grigore T. Popa” University of Medicine and Pharmacy, 700115 Iasi, Romania; anca.ouatu@umfiasi.ro (A.O.); paula-cristina.morariu@umfiasi.ro (P.C.M.); alexandru.oancea@umfiasi.ro (A.F.O.); godun.maria-mihaela@d.umfiasi.ro (M.M.G.); diana-elena.iov@d.umfiasi.ro (D.-E.F.); marius-traian-dragos.dm-marcu@umfiasi.ro (D.T.M.); floria.mariana@umfiasi.ro (M.F.); 4 Department of Internal Medicine, Sf. Spiridon County Clinical Emergency Hospital, 700111 Iasi, Romania; 5 Department of Surgery, Faculty of Medicine, “Grigore T. Popa” University of Medicine and Pharmacy, 700115 Iasi, Romania; livia.baroi@umfiasi.ro; 6 Department of Internal Medicine and Gastroenterology, Carol Davila University of Medicine and Pharmacy, Central Military Emergency University Hospital, 010825 Bucharest, Romania; silviu.stanciu@umfcd.ro; 7 Discipline of Forensic Medicine, Faculty of Medicine, “Grigore T. Popa” University of Medicine and Pharmacy Iasi, 16 Universitatii Street, 700115 Iasi, Romania; anton.knieling@umfiasi.ro

**Keywords:** systemic lupus erythematosus, arrhythmias, atrial fibrillation, pericarditis, myopericarditis, Libman–Sacks endocarditis, coronary artery disease, fibrosis, epidemiology, pathophysiology, endothelial dysfunction, inflammation, csDMARDs, bDMARDs

## Abstract

**Background:** Cardiovascular implications in systemic lupus erythematosus (SLE) are common and varied, including impacts on the pericardium, myocardium, valves, coronary arteries, and conduction system; all of these could be potential substrates or triggers of cardiac arrhythmias by interfering with disease severity and specific medication. Therefore, this narrative review aimed to assess the cardiac involvement in SLE underlying, mainly, cardiac arrhythmias. **Methods:** We analyzed studies, published between 2015 and 2025 on PubMed, which explore cardiovascular involvement with a focus on arrhythmias in SLE from the perspectives of epidemiology, underlying mechanisms, diagnostic techniques, and the impact of standard and biologic therapies. **Results:** The cardiac manifestation of LES (lupus pericarditis, lupus myocarditis, Libman–Sacks endocarditis, coronary artery disease, coronary vasculitis or myocardial fibrosis) represents a substrate for arrhythmia risk. These substrates, in association with other arrhythmias mechanisms considered as triggers or conduction abnormalities, determined arrhythmogenic conditions in these patients. In addition to structural heart disease, arrhythmias in SLE are caused by ongoing inflammation, immune system irregularities, microvascular problems, autonomic imbalance, oxidative stress, and side effects from treatments. Despite this complex background, arrhythmias are often overlooked and not routinely investigated in SLE care. Data that show how disease-modifying drugs may affect arrhythmias are limited and inconsistent, highlighting significant gaps in knowledge. Cardiac arrhythmias are a significant but, as yet, insufficiently underrecognized aspect of SLE, with serious implications for prognosis. **Conclusions:** Systemic lupus erythematosus causes cardiovascular involvement that is associated with arrhythmias through various and complexes mechanisms, mainly related to direct cardiovascular structural damage, systemic inflammation or specific therapies. Data on arrhythmias secondary to cardiovascular damage in patients with SLE in the literature are limited. Therefore, early detection of electrical issues, regular cardiovascular evaluation in high-risk patients, and careful management of treatment effects are vital. A coordinated, multidisciplinary cardio-rheumatology approach is essential to improving arrhythmia detection, tailoring treatments, and ultimately decreasing cardiovascular complications and deaths in SLE patients.

## 1. Introduction

Systemic lupus erythematosus (SLE) is a long-term autoimmune disorder characterized by the production of autoantibodies and the formation of immune complexes, which result in persistent tissue damage and extensive inflammation. Its clinical presentation is highly variable, covering a range of laboratory, clinical, and histopathological features that demonstrate its ability to affect multiple organ systems, including the skin, joints, kidneys, lungs, nervous system, and cardiovascular (CV) system. The disease often follows a relapsing–remitting pattern, with periods of flare-ups and remission. Despite the improvement in outcomes through corticosteroids and advanced immunomodulatory treatments, patients with SLE continue to face higher mortality rates [1].

Cardiac involvement in SLE can affect the pericardium, myocardium, cardiac valves, and coronary arteries, becoming a major contributor to both morbidity and mortality. Patients with SLE have more than twice the risk of developing cardiovascular disease (CVD) compared with the general population. A range of arrhythmias (atrioventricular block, bundle branch block, left anterior fascicular block, and sick sinus syndrome) has been documented, highlighting immune-mediated injury to the cardiac conduction system. The electrocardiogram (ECG) is a non-invasive, widely accessible diagnostic modality for identifying arrhythmias, ischemia, myocardial involvement, and structural abnormalities, often showing characteristic ECG changes that indicate active damage to the myocardium and conduction system [2].

Due to the heterogeneous nature of SLE, historically referred to as “the great imitator” or “the disease with a thousand faces,” and given its capacity to affect multiple organ systems, a multidisciplinary approach is essential for comprehensive patient care. Collaboration among rheumatologists, cardiologists, internal medicine specialists, and other physicians is particularly important for the early identification, assessment, and management of arrhythmias.

This narrative review provides a comprehensive overview of cardiac arrhythmias in SLE, an often overlooked but clinically relevant complication at the intersection of rheumatology and cardiology. Adopting a dual rheumatology–cardiology perspective, the review synthesizes current evidence of CV involvement in SLE mainly focused on cardiac arrhythmias (with epidemiology, pathophysiological mechanisms, diagnostic and treatment strategies) including CV implications of antirheumatic therapies. By underscoring the multifactorial nature of arrhythmogenesis in SLE, it emphasizes the importance of interdisciplinary collaboration for early detection, personalized management, and optimization of CV outcomes, ultimately aiming to improve long-term patient prognosis.

This narrative review identifies CV involvement with a focus on the arrhythmias in patients with SLE. To the best of our knowledge, there is currently no systematic review or meta-analysis focusing specifically on arrhythmias in patients with SLE. Therefore, the literature search was conducted using the PubMed database and included the systematic reviews from the last ten years about CV involvement in SLE aiming: (1) to describe the main CVD associated with LES and (2) to identify the current evidence on the epidemiology, pathophysiology, diagnosis, and management of arrhythmias (identified, mainly, via electrocardiographic abnormalities) in these CVDs associated with LES.

## 2. Cardiac Involvement in Systemic Lupus Erythematosus

Cardiac involvement persists as a critical clinical challenge despite advances in immunosuppressive therapy. The CV risk among SLE patients remains disproportionately high, ranging from 9- to 50-fold higher than that observed in age- and sex-matched populations. This excess risk manifests across a diverse clinical spectrum, from silent ECG changes to acute life-threatening events, reflecting the complex interaction of traditional risk factors, chronic inflammation, immune dysregulation, and treatment-related toxicities that characterize CVD pathogenesis in SLE [3]. A single-center retrospective study of 390 SLE patients (mean age 36.5 years, 90.9% female) found that 45.2% developed CV complications, most commonly hypertension (22.4%), valvular heart disease (13.2%), and dyslipidemia (9.2%), with heart failure, pericarditis, and ischemic heart disease being less frequent. These findings underscore that CV implications in SLE extend beyond atherosclerotic disease, highlighting the importance of regular screening, especially in patients with renal involvement or high-risk immunologic profiles [4].

### 2.1. Pericarditis and Myopericarditis

**Pericarditis**, by definition, is an inflammation of the serous sac surrounding the myocardium; it is the most common cardiac complication in SLE, affecting around 25% of patients at some stage of their disease. Some post mortem studies report a prevalence as high as 62%, though it is unusual for pericarditis to be the only manifestation. The condition’s symptomatology can vary widely, from mild chest pain that worsens when the patient lies flat and improves when leaning forward to severe, debilitating pleuritic chest pain localized to the substernal or precordial area, reduced heart sounds, fever, tachycardia, and shortness of breath. Intriguingly, pericardial tamponade was observed as the initial presentation in 30% of SLE-related pericarditis cases across several studies [5].

To define pericarditis, the Safety Estrogens in Lupus Erythematosus National Assessment–SLE Disease Activity Index (SELENA-SLEDAI) requires at least one of the following criteria: typical pericardial chest pain, a pericardial friction rub on auscultation, evidence of pericardial effusion on imaging, or electrocardiographic findings consistent with pericarditis [6]. Case reports have shown that SLE pericarditis often presents with characteristic ECG changes (diffuse ST-segment elevations or PR-interval depressions). It prompts further evaluation for an autoimmune etiology and is typically confirmed by positive anti-dsDNA, anti-Smith, and anti-Sm/RNP antibodies [7].

Recurrent pericarditis was interpreted as a new episode occurring at least six weeks after the initial episode, with multiple recurrences noted when supplementary episodes occurred at similar intervals. In a cohort of 590 patients with pericarditis, 20.3% experienced recurrence, primarily within the first year. The principal risk factors included younger age, higher SLEDAI scores indicating greater disease activity, and increased oral prednisone doses [6].

Cardiac arrhythmias in the context of pericardial syndrome have been poorly investigated. Isolated atrioventricular blocks are estimated to have a prevalence of approximately 5%, with third-degree atrioventricular block appearing to be even rarer. Autopsy studies in patients with SLE have revealed fibrosis of the atrioventricular node and conduction system, periarteritis affecting the nodal arteries, and sinus node involvement during episodes of active pericarditis [8]. Therefore, the known mechanisms linked to sinus node dysfunction are based on immune-mediated injury to conduction tissue, vasculitis affecting small vessels, and periarterial inflammation of the sinus nodal arteries, as well as cardiotoxic effects from antimalarial medications [9].

**Lupus myocarditis (LM)** is an uncommon, potentially severe manifestation of SLE, affecting approximately 5–10% of patients [10]. In a study by Borenstein et al., only 5 cases of myocarditis were identified among 140 patients with SLE. Contrariwise, Badui et al. reported a higher incidence (14%) in a prospective study, and Cheng et al. documented what is believed to be the first case of a patient presenting with severe left ventricular dysfunction without any other clinical features of SLE [11].

A multicenter retrospective study of 637 patients with SLE identified 25 cases of LM (3.9%). Most affected patients were women (72%), and myocarditis typically developed a median of one year after SLE diagnosis. Clinical manifestations included chest pain or dyspnea in nearly half of patients, while 20% were asymptomatic. Compared with non-SLE myocarditis, LM patients had more conduction abnormalities (22% vs. 5%) and more frequent reductions in left ventricular ejection fraction to <55%. They also showed increased inflammatory markers and elevated pro-BNP levels. Serologically, antiphospholipid syndrome (APS) was more common (36% vs. 10%), and anti-β2-glycoprotein I antibodies were independently associated with myocarditis (HR 2.31; *p* = 0.014) and with a higher number of active British Isles Lupus Assessment Group domains (HR 1.46; *p* = 0.003). Overall, the study demonstrates that LM manifests with distinct clinical, serologic, and histologic characteristics that are closely associated with systemic disease activity and the presence of antiphospholipid antibodies (APAs) [12]. One meta-analysis of 25 studies including 8089 patients with SLE showed that APAs significantly increased the risk of heart valve disease (OR 2.24, 95% CI 1.58–3.18). Lupus anticoagulant (LAC) conferred the highest risk (OR 4.90), followed by anticardiolipin (OR 2.69) and anti-β2 glycoprotein I antibodies (OR 1.70). These results support routine cardiac surveillance in aPL-positive SLE patients [13].

Myocardial involvement is frequently observed but often remains subclinical in SLE, even if can be detectable in up to 22–47% of patients, depending on the imaging modality. Cardiac magnetic resonance (CMR) is the most sensitive non-invasive method for analyzing active inflammation (T2-weighted imaging and early gadolinium enhancement) and chronic damage, such as necrosis or fibrosis (late gadolinium enhancement). CMR-detected myocardial injury correlates with higher disease activity and anti-dsDNA positivity, highlighting the connection between systemic autoimmunity and cardiac damage. Echocardiographic findings, such as reduced Tricuspid Annular Plane Systolic Excursion, increased indexed left ventricular diameter, impaired global longitudinal strain, and abnormal wall motion, also correlate with CMR results and may serve as predictive markers when CMR is unavailable [14].

Myocarditis can progress to arrhythmias, conduction abnormalities, dilated cardiomyopathy, and heart failure, all of which require prompt identification and appropriate management. Primary LM is considered rare, and only a small number of cases describe acute myocarditis and heart failure as early presentations of SLE [11]. Diagnosis is often made on clinical suspicion, particularly when myocardial dysfunction or unexplained arrhythmias are present. Non-specific ECG abnormalities are frequently observed, occurring in up to 70% of patients with LM. The most common findings include sinus tachycardia (75%) and non-specific ST-segment and T-wave changes (70%). Arrhythmias are reported in approximately 20% of cases and may include ventricular extrasystoles, atrial fibrillation (AF), and ventricular tachycardia. Despite its limited specificity, ECG remains valuable for excluding alternative causes of myocardial impairment, such as myocardial infarction, hypertensive heart disease, or chloroquine-induced cardiomyopathy. These differential diagnoses should be carefully considered, particularly in patients with long-standing SLE or additional CV risk factors [14].

In refractory LM, rituximab has proven to be a promising salvage therapy. A single-center retrospective study of 13 Chinese female SLE patients with LM, drawn from a cohort of 802, found that three patients with corticosteroid-refractory LM responded well to rituximab. All three showed marked clinical improvement, with recovery of cardiac function (confirmed by echocardiography and cardiac MRI) and reduced lupus disease activity [15].

### 2.2. Libman–Sacks Endocarditis (Non-Bacterial Verrucous Endocarditis)

**Libman–Sacks endocarditis (LSE)** is a form of non-bacterial thrombotic endocarditis occurring in about 10% of patients with SLE. Vegetations on the heart valves consist of aggregates of fibrin and platelets in the absence of bacterial infection. Although LSE is rarely associated with valve dysfunction, it typically involves the left-sided valves, particularly the mitral and aortic valves, though other valves may also be affected. Underlying endothelial damage is linked to the hypercoagulable state often seen in SLE, APS, and certain neoplasms, suggesting that anticoagulant therapy should be considered for all patients diagnosed with LSE. There is a strong association between APS and LSE. Recent retrospective cohort studies have shown that patients with LSE are more likely to have β2GP1 IgG antibodies and LAC [16].

Diagnosing LSE requires strong clinical suspicion because no specific laboratory test confirms the condition. Although pathological examination can establish the diagnosis, clinicians typically rely on imaging, particularly echocardiography. Transesophageal echocardiography offers significantly greater sensitivity (80–90%) than transthoracic imaging for detecting LSE vegetations. Diagnosis is supported by echocardiographic findings, immunologic testing, and histologic features when available. To distinguish LSE from infective endocarditis, clinicians must evaluate blood counts, metabolic panels, and obtain blood cultures, alongside a hypercoagulable assessment (LAC and APAs). The absence of pathogenic microorganisms helps favor a diagnosis of non-bacterial thrombotic endocarditis rather than infective endocarditis [17].

In a cross-sectional Doppler echocardiography study of 342 SLE patients (297 women and 45 men), Moyssakis et al. identified 38 LSE patients, most commonly on the mitral valve (24 cases), followed by the aortic valve (13 cases), and rarely on the tricuspid valve (1 case). The study found that roughly 10% of SLE patients had Libman–Sacks lesions, which correlated with longer disease duration, higher disease activity, and the presence of APAs. These findings underscore endocarditis as a critical potential cardiac manifestation in SLE [18].

The mechanisms linking LSE and arrhythmias in SLE remain poorly understood. The literature suggests that subendocardial inflammation and thrombosis may contribute to cardiac electrical instability. Proposed mechanisms for LSE formation include: (1) immune-mediated endothelial damage with microthrombus development; (2) deposition of immunoglobulins and complement; (3) hypercoagulability related to SLE disease activity and antiphospholipid syndrome. Current evidence indicates that systemic autoimmune diseases affect the heart through tissue damage, immune complex deposition, complement activation, and endothelial cell activation [19]. Using the National Inpatient Sample (2019–2020), this study analyzed 150,411 hospitalized adults with SLE. Although not statistically significant, patients with SLE and LSE were more likely to have AF than those without LSE (OR 4.45, CI 0.77–25.57, *p* = 0.10), suggesting a possible trend towards increased arrhythmic risk [20].

Management of non-bacterial thrombotic endocarditis in the context of systemic autoimmune disease is fundamentally directed at controlling the underlying pathology. In conditions such as SLE and APS, targeted immunomodulatory strategies are essential, as effective suppression of systemic inflammation reduces the risk of valvular injury, thrombus formation, and subsequent CV complications. Standard measures (statin therapy, antihypertensive treatment, antiplatelet agents such as aspirin, and lifestyle modification) remain integral to long-term management. Together, these strategies aim to mitigate disease-related inflammation and thrombosis, thereby lowering the risk of morbidity from both infective and non-bacterial thrombotic endocarditis [17]. A retrospective study comparing 11 SLE patients with LSE to 29 SLE controls found that, although overall disease activity was comparable between groups, APA prevalence, particularly triple positivity, was markedly higher in LSE patients (72.7% vs. 13.8%). Despite LSE lesions persisting for approximately five years during follow-up, no patients required surgical valve intervention. Treatment strategies centered on anticoagulation combined with intensified immunosuppression. Notably, the sole patient not receiving anticoagulation experienced a cerebral infarction. The findings underscore the robust link between LSE and APAs, emphasizing the importance of surveillance and anticoagulation therapy to mitigate thromboembolic complications [21].

### 2.3. Coronary Artery Disease

**Coronary artery disease (CAD)** is reported for nearly one-third of all deaths among patients with SLE. Premenopausal women with SLE have an extraordinary elevation in the risk of acute myocardial infarction, reportedly up to 50 times higher than age-matched controls, with the first year after diagnosis being the most critical period incidence. The underlying mechanism of acute coronary syndrome in these patients is atherosclerosis, which has been observed in about half of cases in autopsy studies. Less frequently, acute coronary syndrome results from alternative pathologies such as thrombosis, vasculitis, or spontaneous coronary artery dissection [22].

Accelerated atherosclerosis that occurs in SLE patient results from immune-driven inflammation and involves three mechanisms. First, endothelial injury is caused by reduced endothelial progenitor cells and low VEGF levels. Pro-inflammatory cytokines (TNF-α, IL-1) upregulate adhesion molecules, promote LDL oxidation and foam cell formation, and decrease nitric oxide synthase activity, collectively impairing endothelial function. Second, innate immune activation via TLR7 and TLR9 increases type I interferon production, enhances foam cell formation, and triggers platelet activation and thrombosis, intensifying vascular inflammation. Third, adaptive immune dysregulation through Treg/Th17 imbalance, pro-inflammatory Th1/Th17 activity, CD4+ T-cell infiltration, and pathogenic autoantibodies accelerates plaque growth and instability. Together, these mechanisms create a sustained pro-inflammatory, pro-atherogenic environment, promoting early and severe atherosclerotic disease characteristic of SLE [23].

Patients with SLE have a high burden of CV risk due to both traditional and disease-specific factors. Additionally, insulin resistance, diabetes, systemic hypertension, smoking, and dyslipidemia contribute to coronary calcification and are amplified by SLE’s chronic pro-inflammatory environment [24].

The clinical expression of CAD in SLE is highly variable, ranging from exercise-induced angina and impaired functional status to acute myocardial infarction and sudden cardiac death. Accurately assessing CV risk in this population remains challenging, as most conventional calculators, including Globo Risk, SCORE, and ASCVD, are validated only for individuals age over 40 years, and consistently underestimate risk in younger SLE patients [25].

The QRISK3 model incorporates autoimmune disease and corticosteroid use, offering a more appropriate 10-year prediction of myocardial infarction and stroke. Additionally, the SLICC/ACR Damage Index (SDI), which reflects cumulative organ injury, has demonstrated predictive value for 10-year mortality and is associated with CV events when ≥1 [26]. SDI captures permanent damage across 12 organ systems: ocular (0–2), neuropsychiatric (0–6), renal (0–3), pulmonary (0–5), CV (0–6), peripheral vascular (0–5), gastrointestinal (0–6), musculoskeletal (0–7), skin (0–3), endocrine (0–1), gonadal (0–1), and malignancies (0–2), with a theoretical maximum score of 47 [27]. Except for acute events like myocardial infarction and stroke, which are noted at their onset, all other items must persist for at least six months. It is important to note that damage scores can only remain stable or increase over time [28].

Activity markers and imaging modalities play an important role in identifying subclinical CV disease in SLE. Biomarkers such as C-reactive protein and homocysteine may reflect disease activity, while APAs remain well-established independent predictors of CV risk. Imaging results provide additional diagnostic insight: coronary CT angiography consistently reveals a higher burden of subclinical and multivessel CAD in SLE patients, especially women, where coronary plaque is linked to worse clinical outcomes. Coronary calcium scoring further refines risk assessment by estimating residual CV risk, especially in patients with severe early disease or lupus nephritis, who have higher mortality rates. Collectively, these data support the use of early imaging, including coronary calcium measurement at the time of diagnosis, to improve the detection of subclinical CV involvement in SLE [23].

The management of traditional CV risk factors follows standard prevention guidelines in SLE patients. Hydroxychloroquine (HCQ) has been proven to reduce thrombotic events. However, glucocorticoid doses should be kept as low as possible. While statins might offer vascular protection, the evidence for their universal use remains inconsistent, whereas acetylsalicylic acid provides benefits for patients with established CV risk. The mechanisms underlying CV complications in SLE complicate the development of targeted prevention strategies, and autoimmunity-related vascular injury remains a promising area for future therapies [23].

A Danish registry-based study of 3411 patients with SLE, followed for a median of 8.5 years, demonstrated that SLE is associated with substantially increased long-term CV risk, which includes myocardial infarction, heart failure, and arrhythmias. Notably, the incidence of AF/flutter and serious ventricular arrhythmic events (cardiac arrest and implantable cardioverter–defibrillator placement) was higher in patients with SLE than in matched controls. These facts highlight the arrhythmogenic impact of CAD in this population. Coronary ischemia and myocardial scarring, compounded by SLE-related inflammation, oxidative stress, and microvascular dysfunction, seem to provide a substrate for both atrial and ventricular arrhythmias. These findings indicate that CAD in SLE is not merely a vascular complication but a significant contributor to electrical instability, emphasizing the importance of early CV risk assessment, vigilant arrhythmia screening, and integrated cardio-rheumatologic care [27].

### 2.4. Myocardial Fibrosis and Cardiomyopathy as Arrhythmic Substrates

Lupus cardiomyopathy remains an imprecisely characterized entity and presents significant diagnostic challenges. Nevertheless, its prompt recognition in patients with SLE is crucial, as early detection may help prevent serious cardiac arrhythmias. Longitudinal studies show that CMR-LGE–detected myocardial scars significantly predict sudden cardiac death, arrhythmias, and heart failure. A study was conducted to determine whether scar-imaging echocardiography using an ultrasound multi-pulse scheme (eSCAR) can identify subclinical myocardial involvement in patients with SLE. All participants underwent eSCAR and speckle-tracking echocardiography, including assessment of global longitudinal strain. SLE patients also underwent evaluation of disease activity and were followed prospectively for 12 months. Myocardial scars were detected using eSCAR in 19% of SLE patients, almost exclusively within the infero-septal segments, and in none of the controls. Global longitudinal strain values were significantly reduced across multiple myocardial regions in patients with SLE compared with controls, with the most significant impairment observed in the infero-septal segments. eSCAR-positive patients exhibited higher cumulative and current prednisone exposure, as well as significantly elevated anti-dsDNA antibody levels. Furthermore, the presence of eSCAR-detected scarring was associated with a substantially increased risk of SLE flares during follow-up (hazard ratio 4.91; 95% CI 1.43–16.83; *p* = 0.0001). In summary, infero-septal myocardial scarring was identified in approximately one-fifth of SLE patients. Subclinical myocardial involvement detected via eSCAR was associated with glucocorticoid exposure, elevated anti-dsDNA antibodies, and a higher likelihood of subsequent SLE flares [29].

One study aimed to assess the prevalence of myocardial fibrosis detected using CMR in a cohort of patients with SLE and to examine the relationship between CMR-derived fibrosis markers and biochemical or clinical SLE-related risk factors. Particular attention was given to LAC, which our group has previously linked to myocardial remodeling and impaired diastolic function. Overall, SLE patients demonstrated CMR evidence of myocardial fibrosis, with these abnormalities closely linked to the presence of LAC. These findings reinforce the pathophysiological role of LAC as a contributor to microvascular dysfunction and subsequent myocardial injury in SLE [30]. A systematic review and meta-analysis of nine studies found that patients with SLE have significantly impaired myocardial function on speckle-tracking echocardiography compared with controls. These findings indicate subclinical biventricular myocardial dysfunction in SLE [31].

Using machine learning techniques, three crucial genes associated with SLE and AF were identified: TMEM45A, ITGB2, and NFKBIA. These outcomes indicate that TMEM45A stimulates cardiac fibroblast fibrosis, suggesting its contribution to the development of AF. GSEA analysis of TMEM45A within the AF dataset revealed improvement of the TGF-β signaling pathway. These results support the notion that TMEM45A may contribute to cardiac fibrosis by modulating the TGF-β signaling pathway. As a transmembrane protein, TMEM45A typically exerts its biological functions by interacting with specific ligands. Therefore, TMEM45A was experimentally confirmed to have a pro-fibrotic effect both in vivo and in vitro, underscoring its promise as a potential therapeutic target for AF in SLE patients [32].

### 2.5. Coronary Artery Vasculitis and Microvascular Disease

Vasculitis, defined by inflammatory cell infiltration leading to blood vessel wall necrosis, is a hallmark pathological process in SLE progression and ranks among the principal causes of mortality in patients with established disease. The clinical presentation of this vascular inflammation varies considerably based on vessel caliber (arteries, veins, and/or capillaries) and anatomical location (cutaneous versus visceral involvement), resulting in a prognostic spectrum that extends from benign manifestations to potentially fatal complications [33]. It occurs in 11–36% of patients and is most commonly observed during active disease. Cutaneous vasculitis is the predominant form, accounting for up to 69% of vasculitis manifestations [34]. Lupus vasculitis may be linked to APS, which is characterized by the presence of antiphospholipid antibodies [35].

Coronary artery vasculitis is exceptionally uncommon, with only sporadic case reports available in medical literature. Its presence typically indicates severe disease activity and may be accompanied by systemic symptoms, including fever, fatigue, anemia, elevated inflammatory markers, livedo reticularis, and weight loss. However, there are documented cases of coronary vasculitis occurring without any clinical or serological signs of active SLE [34].

The literature does not provide specific studies or well-documented case reports directly demonstrating the mechanisms by which coronary artery vasculitis in SLE leads to arrhythmias. However, immune complex deposition and complement activation in SLE induce inflammatory changes in the coronary arteries, resulting in luminal narrowing, vasospasm, and thrombosis. These vascular alterations may impair myocardial perfusion, particularly to the cardiac conduction system, including the sinoatrial node, atrioventricular node, and Hiss–Purkinje network. Consequently, myocardial ischemia and electrical instability may occur, providing a plausible mechanistic basis for the contribution of coronary artery vasculitis to arrhythmogenesis in SLE [36].

Diagnosis is typically established via sequential coronary angiography demonstrating arterial aneurysms, progressive stenotic narrowing, and/or acute arterial occlusions. Histopathological examination reveals coronary artery thrombosis or immune complex deposition, with lymphocytic and neutrophilic infiltration and fibrinoid necrosis. In previous cases, pathological examination has shown that inflammation produces segmental stenoses, resulting in ectasia or aneurysm formation. One case showed lymphocytic infiltration in the coronary artery intima along with severe diffuse sclerosis of the major epicardial vessels, resembling transplant-associated vasculopathy but notably sparing the small branch arteries. The vasculopathy did not show the calcification or atherosclerotic changes typical of CAD. Instead, it showed a concentric thickening pattern, which is characteristic of coronary vasculitis. In the absence of other causes, this was attributed to chronic inflammation associated with SLE [37].

No standardized treatment protocol exists for coronary vasculitis in SLE, though limited case reports have shown favorable outcomes with high-dose intravenous methylprednisolone and intravenous cyclophosphamide in patients with active disease. In cases of low response to immunosuppressive therapy, orthotopic heart transplantation should be considered as a therapeutic option. Timely diagnosis and appropriate management are decisive. Treatment strategies must be individualized according to clinical presentation and disease severity. Since there are no randomized controlled trials specifically on lupus vasculitis, treatment strategies are based on evidence from other autoimmune diseases, different vasculitis syndromes, or individual case reports [35].

## 3. Arrhythmias in Systemic Lupus Erythematosus

### 3.1. Epidemiology of Cardiac Arrhythmias in Systemic Lupus Erythematosus

SLE is an autoimmune disease associated with an increased risk of CV complications, which account for approximately 10–15% of SLE-related deaths. However, data on the association between arrhythmias and SLE are limited, and existing research findings remain inconsistent and controversial [38].

Sinus tachycardia, AF and atrial ectopic beats are the most commonly observed arrhythmias in patients with SLE. These arrhythmias are often transient and may be associated with LM or disease flares, particularly when accompanied by fever, volume depletion, or congestive heart failure. Some studies have reported that sinus tachycardia occurs in up to 50% of patients with SLE, and in certain cases, it is the sole cardiac manifestation of the disease. In a study conducted by Teixeira et al. involving 317 patients with SLE, Holter monitoring detected abnormalities in about 85% of cases. The most frequent findings were supraventricular ectopy (63.4%), followed by atrial tachycardia (15.5%) and AF (2.8%) [39]. Focal atrial tachycardia is frequently reported in patients with SLE. It has been proposed that systemic inflammation in lupus may promote arrhythmias by exerting direct inflammatory effects on cardiac valves [5].

A meta-analysis revealed that the incidence of AF in SLE patients reaches 1.15%, and confirmed that the prevalence of AF events is significantly higher in SLE cohorts [39]. The prevalence of AF in SLE may be influenced by geographic variation. A previous systematic review and meta-analysis examined whether SLE is associated with an increased risk of AF. Six cohort studies, comprising 311,844 participants (78,134 with SLE and 347,883 controls), were included. The pooled results indicated that SLE was associated with a higher risk of AF (RR = 1.85; 95% CI: 1.23–2.79), although significant heterogeneity was present (I^2^ = 96%). Subgroup analysis of four studies from European and American populations demonstrated low heterogeneity (I^2^ = 9%) and a significant association between SLE and AF (RR = 1.79; 95% CI: 1.61–1.98). In contrast, two Korean studies showed high heterogeneity (I^2^ = 98%) and no clear association. This research concluded that SLE may be a risk factor for AF, and the strength of this association appears to vary by geographic region [38].

Another study analysed the 2016–2019 National Inpatient Sample to evaluate the impact of AF on inpatient outcomes among adults hospitalised with SLE. Of 41,004 SLE hospitalizations, 3.65% had a concurrent diagnosis of AF. Compared with SLE patients without AF, those with AF had significantly worse outcomes. AF was associated with higher in-hospital mortality (aOR 2.07) and longer hospital stays. Additionally, AF was linked to increased risks of non-ST-elevation myocardial infarction, pericardial effusion, cardiac tamponade, and cardiogenic shock. AF in hospitalised SLE patients correlates with notably worse clinical outcomes, including higher mortality, complications, and healthcare burden [40].

Although supraventricular tachycardia is the most frequently observed arrhythmia in SLE, ventricular arrhythmias can also occur. Ventricular tachycardia may be the initial clinical presentation of acute myocarditis in patients with SLE. The presence of anti-Ro/SSA antibodies has been associated with a higher risk of QTc prolongation, a known risk factor for ventricular arrhythmias and sudden cardiac death. These patients also tend to present reduced heart rate variability and a higher incidence of ventricular late potentials, further suggesting underlying electrical instability [39].

Severe brady-arrhythmias are rare in SLE. One study analysed data from 1366 patients in the Toronto Lupus Clinic to describe such cases. In total, 18 patients (1.32%) received a permanent pacemaker: 13 (0.95%) for complete atrioventricular block and 5 (0.37%) for sick sinus syndrome. Compared to matched controls, patients with a permanent pacemaker had higher rates of coronary artery disease, hypertension, dyslipidaemia, and longer antimalarial therapy. They also had increased prevalence of first-degree atrioventricular block, right bundle branch block, left anterior fascicular block, and septal hypertrophy. In addition to traditional risk factors, prolonged antimalarial therapy might lead to severe brady-arrhythmias in SLE patients [41].

While ventricular arrhythmias and conduction abnormalities are infrequently described in SLE, a nationwide Danish study using administrative registries evaluated these risks. The cohort included SLE patients diagnosed between 1996 and 2018 who had no prior CVD. Over 10 years, SLE patients had an absolute risk of 4.35% (95% CI: 3.61–5.18%) for AF or flutter, 0.89% (95% CI: 0.58–1.31%) for the composite outcome of implantable cardioverter–defibrillator implantation, ventricular arrhythmia, or cardiac arrest, and 0.59% (95% CI: 0.36–0.94%) for the composite of pacemaker implantation, atrioventricular block, or sinoatrial dysfunction [27].

Abnormalities in the cardiac conduction system and other arrhythmias (Table 1) have been relatively understudied, and, as a result, their prevalence and the factors that contribute to these rhythm disturbances are still not well understood [41].

### 3.2. Pathophysiological Mechanisms of Arrhythmias

SLE induces a complex interplay of immune dysregulation and chronic systemic inflammation, predisposing patients to arrhythmias through multiple interrelated mechanisms. Immune complex deposition in myocardial tissue and complement activation trigger local inflammatory cascades, leading to myocyte injury and apoptosis, interstitial fibrosis, and altered extracellular matrix composition. These structural changes create heterogeneous conduction pathways that favor re-entrant arrhythmias [38].

Pro-inflammatory cytokines including TNF-α, interleukin-1 beta (IL-1β), and interleukin-6 (IL-6) are crucial in electrophysiological remodeling. They influence ion channel expression and function by decreasing sodium, potassium, and calcium channel activities. This results in either shortened or prolonged action potential durations based on local myocardial conditions, increased dispersion of repolarization, and the development of early after-depolarizations [42]. Oxidative stress, caused by reactive oxygen species produced in response to cytokine activity, further impairs ion channel function and gap junction coupling, increasing conduction heterogeneity and susceptibility to arrhythmias [38].

Chronic inflammation also promotes fibroblast activation and myocardial collagen deposition, resulting in atrial and ventricular fibrosis. Fibrosis interrupts electrical continuity and enhances the formation of re-entrant circuits, particularly in the atria, predisposing patients to AF [42]. Renin–angiotensin–aldosterone system activation in SLE patients contributes further, with angiotensin II and aldosterone inducing TGF-β1–mediated profibrotic signaling, oxidative stress, and inflammation, which collectively drive structural remodeling, hypertrophy, and arrhythmogenic substrate formation [43].

Autonomic nervous system dysfunction is another critical mechanism. Sympathetic overactivity, frequently observed in chronic autoimmune inflammation, alters calcium handling and action potential propagation, while parasympathetic dysregulation can prolong atrial refractoriness, facilitating ectopic firing and re-entry [38]. Electrolyte imbalances, particularly fluctuations in potassium, calcium, and magnesium, further destabilize membrane excitability and conduction velocity, increasing the likelihood of ventricular tachycardia and brady-arrhythmias [44].

Arrhythmogenesis linked to SLE is further worsened by the presence of additional CV pathology. Myocarditis and pericarditis produce local inflammation, impair myocardial contractility, and increase pericardial pressure, all of which disrupt normal electrical conduction. The hypercoagulable state associated with APAs can contribute to microvascular ischemia, which exacerbates electrical instability. Epidemiological evidence supports these mechanistic pathways: in silico studies demonstrate that severe inflammation in SLE can induce atrial re-entrant circuits and conduction blocks, while large-scale cohort studies show strong associations between systemic inflammatory markers and the incidence of AF, ventricular arrhythmias, and brady-arrhythmias [44].

A systematic review and meta-analysis of 123 studies evaluated endothelial cell marker dysregulation in SLE and its association with disease activity. Significant correlations with disease activity were observed for pentraxin-3, thrombomodulin, VEGF, VCAM-1, ICAM-1, IP-10, and MCP-1, whereas other dysregulated endothelial cell markers were not associated with disease activity. These findings highlight the complex role of endothelial dysfunction in SLE and support further longitudinal studies to clarify its contribution to CV risk [45].

**Figure 1:**
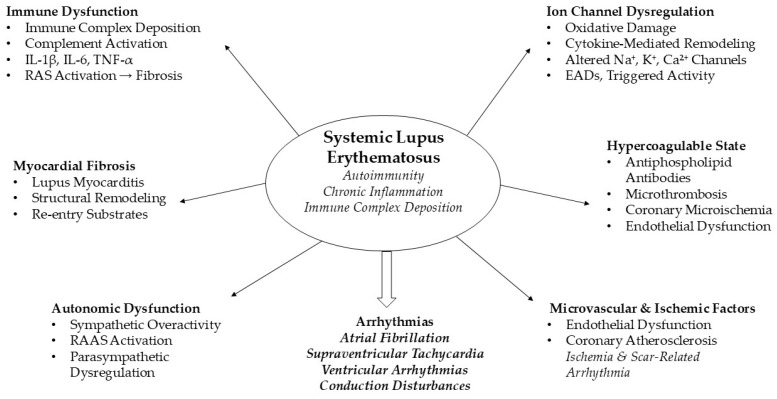
The pathophysiological pathways linking systemic lupus erythematosus (SLE) with arrhythmogenesis. *SLE, Systemic Lupus Erythematosus; DMARDs, Disease-Modifying Antirheumatic Drugs.*

These findings demonstrate a bidirectional and self-reinforcing link between inflammation and arrhythmias in SLE. Immune-driven myocardial damage, ion channel issues, oxidative stress, structural changes, activation of the renin–angiotensin–aldosterone system, autonomic imbalance, and coagulation problems all contribute to both atrial and ventricular arrhythmias. Therefore, therapies targeting inflammation, oxidative stress, and the renin–angiotensin–aldosterone system could be effective in preventing arrhythmic complications in SLE. The major pathophysiological pathways linking SLE to arrhythmogenesis are summarized in Figure 1.

### 3.3. Pro-Arrhythmogenic Relation Between Antibodies and Systemic Lupus Erythematosus

#### 3.3.1. Anti-Ro Antibodies

Anti-Ro/SSA antibodies are associated with four conditions: SLE, primary Sjögren’s syndrome, subacute cutaneous lupus, and congenital heart block. They consist of two subunits: anti-Ro52, which is more commonly seen in SS, and anti-Ro60, which is more typical of SLE. The prevalence of anti-Ro/SSA antibodies ranges from 25–50% in SLE and 40–95% in primary Sjögren’s syndrome, with antibody positivity forming part of the EULAR/ACR classification criteria and carrying significant weight for diagnosis [46]. SLE patients may show ventricular repolarization abnormalities linked to anti-Ro antibodies, reflecting the 2% risk of congenital heart block in babies of anti-Ro-positive mothers [47].

The relationship between ventricular repolarization abnormalities and anti-Ro antibodies was first explored in patients with connective tissue diseases (CTDS), including SLE. In a study of 57 CTDS patients, Lazzarini et al. reported that 58% of anti-Ro positive patients had QTc >440 ms, while none of the anti-Ro negative patients did, and QTc duration correlated with anti-Ro antibody titers. Another study using 24 h ambulatory ECG in 46 CTDS patients confirmed the association between QTc prolongation and anti-Ro positivity. Additionally, anti-Ro-positive patients had a higher incidence of ventricular arrhythmias, attributed to prolonged QTc [47].

In a study of 779 adult SLE patients from 19 SLICC Inception Registry centers, ECG abnormalities were common. Nonspecific ST-T changes occurred in 30.9% of patients and supraventricular arrhythmias in 1.3%. QTc ≥ 440 msec was present in 15.3% and ≥460 msec in 5.3%, while mean QTd was 34.2 ± 14.7 msec, with 38.1% having QTd ≥40 msec. Anti-Ro/SSA level or specificity (52 or 60 kD) was not associated with QTc or QTd, although confidence intervals were wide. Higher total SDI was significantly associated with QTc >440 msec (OR 1.38, 95% CI 1.06–1.79) [48]. In contrast, in a cohort of 145 patients with SLE, anti-Ro/SSA antibodies were present in 32% of cases but were not associated with atrioventricular conduction abnormalities, alterations in ECG intervals (PR, QRS, QTc), clinically significant arrhythmias on 24 h Holter monitoring, heart rate parameters, or echocardiographic measures, indicating no evident impact on cardiac conduction or structural cardiac involvement [49].

The involvement of specific autoantibodies, including anti-SSA/Ro, anti-SSB/La, and anti-RNP, remains controversial. Most available data are derived from uncontrolled studies, isolated case reports, or small case series. Regarding anti-SSA/Ro antibodies, Logar et al. reported a higher prevalence of clinically diagnosed myocarditis and moderate conduction abnormalities (first-degree atrioventricular block, right bundle branch block, and left anterior fascicular block) in anti-SSA/Ro-positive patients compared with anti-SSA/Ro-negative individuals and healthy controls [41].

#### 3.3.2. Antiphospholipid Syndrome

Antiphospholipid syndrome is an autoimmune, thrombo-inflammatory disease characterized by the production of circulating APAs, which play a central role in the blood clot formation in arteries, veins, and small vessels. APAs are a heterogeneous group of immunoglobulins, including LAC, anti-cardiolipin antibodies (aCL), and anti-β2-glycoprotein-I antibodies (anti-β2GPI), directed against phospholipids, cofactors, or phospholipid–cofactor complexes [50]. There is a close clinical and pathophysiological relationship between APS and SLE. Data from the Euro-Phospholipid Project indicate that 36% of APS patients have SLE, while 20–40% of SLE patients possess APAs, and 50–70% of those with APAs progress to definitive APS within 20 years. Additionally, individuals with primary APS may later develop SLE. Secondary APS in SLE patients is associated with increased chronic organ damage and mortality. Moreover, SLE is linked to elevated CV morbidity and thrombosis, driven by both disease-specific and traditional risk factors [51].

A retrospective single-center study included 213 hospitalized patients with paroxysmal AF from Peking University People’s Hospital (January 2012–September 2022), comprising 107 controls and 106 cases. Multivariable analysis showed that APS, aCL positivity, and anti-β2GPI positivity were independent risk factors for AF and differed significantly between AF and non-AF patients. The mechanisms by which APS induces inflammation are complex. Anti-β2GPI can trigger platelet activation and platelet–leukocyte interactions, leading to immune inflammatory responses. Monoclonal APAs can activate neutrophils and induce monocytes to express tissue factors and pro-inflammatory mediators. aCL and anti-β2GPI contribute to myocardial ischemia, myocarditis, and valvular disease, with effects depending on antibody type and titer. Their pathogenic mechanisms involve thrombosis, inflammation, complement activation, and platelet stimulation. Deposition of anti-β2GPI and complement on heart valves can cause inflammation, while molecular mimicry with microbial antigens may promote cardiac injury. Anti-β2GPI may also promote AF through valvular structural changes, and aCL could act similarly, though further studies are needed. Inflammatory and fibrotic processes involving macrophages, mast cells, and Th2 cells, along with cytokines such as TNF, IL-1, CRP, and IL-6, are key in AF development. Thus, APS-related inflammation may overlap with AF inflammatory pathways. Immune activation and inflammation can disrupt atrial electrophysiology, increasing excitability and AF susceptibility. APAs may cause myocardial inflammation and structural remodeling that further predispose to AF [52]. This study is not based on SLE-APS patients but emphasizes the impact of APS on cardiac abnormalities.

### 3.4. Correlation of Cardiac Conduction Abnormalities with Disease Activity in Systemic Lupus Erythematosus

Cardiac involvement in SLE typically presents as pericarditis, while myocarditis and valvular disease are less common. However, conduction system abnormalities and other arrhythmias are less studied, and their prevalence and related factors are not well established [41].

Assessing disease activity in SLE is crucial for evaluating patient outcomes, distinguishing among SLE subgroups, monitoring responses to new therapies, and conducting longitudinal analyses in both observational studies and clinical trials. The SLEDAI-2K is a validated tool that captures the multisystem and fluctuating nature of SLE activity, making it the most widely used disease activity index in clinical practice and research [53]. Introduced in 1992, the SLEDAI scale is widely used to evaluate disease activity in SLE. Over time, several validated adaptations have been developed, including SLEDAI-2K, SELENA-SLEDAI, and Mex-SLEDAI. Among these, SLEDAI-2K (2002) is the most frequently utilized (Table 2). It comprises 24 items (16 clinical and 8 laboratory features), scored for presence within the preceding 10–30 days, irrespective of severity. Unlike the original version, SLEDAI-2K also assigns points for persistent manifestations such as rash, alopecia, mucosal ulcers, and proteinuria >0.5 g/day. The total score ranges from 0 to 105. Using the SLEDAI-2K index, standardized definitions have been established for remission, low disease activity, and high disease activity. Remission is defined as the absence of clinical manifestations of SLE, with or without serological activity, in patients receiving no treatment or only antimalarial therapy. Low disease activity is characterized by an SLEDAI-2K score <3, with a single clinical manifestation scoring 1–2 points, and may be associated with positive or negative serological tests. Patients may be on antimalarials but not on glucocorticoids or other immunosuppressants. High disease activity is defined by a SLEDAI-2K score greater than 6 [54].

Wu et al. examined the association between the SLE Disease Activity Index 2000 (SLEDAI-2K) and ECG abnormalities in a Chinese SLE population. Data were drawn from an SLE database spanning 2018–2023. Weighted multivariable regression and subgroup analyses assessed the independent association between SLEDAI-2K and ECG abnormalities. A total of 317 patients were included. The overall prevalence of ST segment and T wave changes was 37.5%. Analyses revealed a linear relationship between SLEDAI-2K scores and the risk of ST-T changes. Subgroup analyses showed that this association was influenced by female gender, age ≤ 25 years, coexisting autoimmune diseases, and infectious complications. These findings indicate that higher SLEDAI-2K scores are associated with an increased incidence of ST-T changes, suggesting that the SLEDAI-2K may serve as a useful index for detecting early cardiac involvement in SLE patients [55]. Patients with SLE had more frequent non-specific ST-T abnormalities and longer QTc intervals than patients with RA. ST-T abnormalities occurred in 44% of SLE patients versus 17% of RA patients and remained significantly higher after adjustment (adjusted OR 7.8). QTc duration was also longer in SLE by about 26 ms in both unadjusted and adjusted analyses, despite SLE patients being younger and having shorter disease duration [56].

A study of 100 newly diagnosed SLE patients and 100 controls (from 2012–2013) assessed QT interval parameters using a standard 12-lead ECG, measuring QT from Q wave onset to T wave end in lead II or lateral leads (V5, V6). Among 84 patients with high disease activity, QTc > 440 ms occurred in 51 patients versus 6 controls, while prolonged QTd was present in 6 patients and 6 controls. Mean QTc was significantly higher in patients (463.3 ± 27.4 ms) than controls (397.2 ± 31.9 ms; *p* < 0.001), whereas mean QTd was similar (44.4 ± 20.6 ms vs. 39.2 ± 17.7 ms). QTc during severe flares strongly correlated with baseline QTc (r = 0.863), suggesting QTc prolongation may serve as a surrogate marker for SLE disease activity [57]. However, a study from the SLE collaborating clinics (SLICC) cohort showed that 15.3% lupus patients had prolonged QTc, but no association with disease activity was found [47].

Fragmented QRS (fQRS) is a reliable ECG marker of myocardial scarring, characterized by additional notches within the QRS complex, likely caused by altered conduction through tissue affected by ischemia or inflammation. It has diagnostic and prognostic value in conditions such as CAD or cardiac sarcoidosis and is more prevalent in rheumatic diseases. In a study by Hosonuma et al., 44 newly diagnosed SLE patients were evaluated for the association between disease activity and fQRS. The presence of fQRS, detected in 59.1% of patients, was associated with significantly higher SLEDAI-2K scores. These findings suggest that fQRS may indicate subclinical myocardial involvement and serve as a non-invasive marker of increased disease activity in SLE [58].

Patients with SLE have over twice the risk of CAD compared with the general population. Immune-mediated conduction system damage contributes to arrhythmias such as atrioventricular block, bundle branch blocks, sick sinus syndrome, and sinus tachycardia. ECG is a non-invasive tool that helps detect arrhythmias, myocardial injury, and structural abnormalities, with findings often reflecting ongoing cardiac involvement. Reported prevalence includes chronic tachycardia (14.8–18%), AF (0.13–3%), and QT prolongation (15.3–17%) among SLE patients. ST-segment and T-wave abnormalities are also common indicators of myocardial damage. Assessing SLE disease activity is crucial for prognosis, monitoring therapy, and guiding clinical decision making [55].

### 3.5. Impact of Disease-Modifying Antirheumatic Drug (DMARD) Therapy on Arrhythmia Risk in Systemic Lupus Erythematosus

#### 3.5.1. csDMARDs

Conventional synthetic disease-modifying antirheumatic drugs (csDMARDs), including antimalarials such as HCQ, immunosuppressants like azathioprine (AZA), mycophenolate mofetil (MMF), cyclophosphamide (CYC), and methotrexate (MTX), remain the backbone of SLE management. Despite the advent of biologic therapies, csDMARDs are widely used for both induction and maintenance therapy due to their proven efficacy in controlling disease activity, preventing flares, and sparing the use of corticosteroids. HCQ serves as the “anchor drug,” providing immunomodulatory, anti-inflammatory, and cardioprotective effects. Immunosuppressive agents are selected based on organ involvement, disease severity, and patient-specific factors, particularly in renal, hematologic, or severe systemic manifestations. While generally well tolerated, csDMARDs require careful monitoring of hematologic, hepatic, renal, and CV adverse effects. Personalized therapy, consistent monitoring, and patient adherence are essential for achieving optimal long-term results in SLE [59].

Hydroxychloroquine is a cornerstone therapy in SLE for its immunomodulatory, anti-inflammatory, and antithrombotic properties. Clinically, HCQ has been shown to reduce disease activity, prevent flares, and improve long-term survival in patients with SLE. It acts by interfering with lysosomal activity, antigen presentation, and toll-like receptor signaling, thereby modulating both innate and adaptive immune responses. HCQ also demonstrates cardioprotective effects, including reduced thrombosis risk, improved lipid profiles, and mitigation of accelerated atherosclerosis commonly observed in SLE. Additionally, HCQ can lower the risk of renal involvement and may reduce the incidence of neuropsychiatric manifestations. Despite its benefits, long-term use carries potential adverse effects, cardiac toxicity is rare but can manifest as conduction abnormalities or cardiomyopathy, typically requiring biopsy confirmation. Overall, continuous HCQ therapy is associated with sustained clinical benefits, whereas interruption or remote use may diminish protective CV and immunologic effects. Its safety profile and multi-system benefits make HCQ an essential component of SLE management [60]. In a national cohort of 1820 Medicare-insured kidney transplant patients with SLE or scleroderma, the use of HCQ was studied alongside cardiac and transplant outcomes. Compared with standard triple immunosuppression, regimens that included HCQ were associated with a higher risk of abnormal ECG findings, QT prolongation, and ventricular arrhythmias. Overall, HCQ appears to be safe for selected transplant recipients, but close monitoring for cardiac arrhythmias is recommended [61].

Observational studies indicate that HCQ use in SLE is linked to a markedly lower incidence of AF, with one cohort reporting an ~88% risk reduction, and no increase in ventricular arrhythmias. Mechanistically, HCQ may stabilize atrial action potentials and mitigate proarrhythmic remodeling associated with SLE, including fibrosis, immune complex deposition, autonomic dysfunction, and oxidative stress. Population-based studies from Canada, Taiwan, and France further support HCQ’s CV safety, demonstrating no significant rise in arrhythmia risk and a potential reduction in major CV events. These findings support the long-term arrhythmia safety of HCQ and suggest additional protective effects against AF in SLE patients [62]. In retrospective and prospective cohorts of 90 and 84 SLE patients, respectively, HCQ use and blood HCQ levels were not associated with QTc prolongation. QTc intervals did not differ significantly between HCQ-treated and untreated patients, including those with chronic kidney disease. In the prospective cohort, QTc duration was not correlated with HCQ dose, treatment duration, measured blood HCQ levels, renal function, or underlying cardiac abnormalities. These findings indicate that HCQ does not result in clinically meaningful QTc prolongation in patients with SLE [63].

By contrast, in a retrospective cohort of 126 patients with SLE (42 HCQ-treated), HCQ therapy was associated with a significant increase in QTc duration, whereas no significant change was observed in untreated controls. Among patients treated with HCQ who developed QTc prolongation, more often had a longer disease duration and coexisting hypertension. However, in multivariable analysis, these factors were not independently linked to QTc prolongation. These results imply that HCQ might play a role in QTc prolongation in SLE, suggesting that ECG monitoring should be considered, especially for patients with a longer disease history and CV comorbidities [64].

The existing literature provides limited and often vague data, based on superficial evaluation, regarding the association between immunosuppressive therapies (MMF, CYC, AZA) and arrhythmias in SLE. A large US-Medicaid-based study compared CVD risk among SLE patients initiating MMF, CYC, or AZA. Overall, MMF was associated with a modestly lower risk of CVD events than AZA in a 12-month intention-to-treat analysis (HR 0.68; 95% CI 0.47–0.98), although the difference with CYC was not statistically significant. No significant differences in all-cause mortality were observed. These findings suggest that while MMF may offer some short-term CV benefit over AZA, immunosuppressive choice alone does not markedly alter long-term CVD risk in SLE, highlighting the need for comprehensive management that includes disease control, minimization of glucocorticoid exposure, and attention to traditional CV risk factors [65]. Most available data are from isolated case reports, pharmacovigilance signals, or extrapolations from non-SLE populations such as transplant, oncology, and dermatology. These sources suggest that MTX and CYC may rarely cause ventricular arrhythmias, while MMF has been linked to tachycardia or rare cases of AF. However, these observations are not supported by statistically powered, SLE-specific studies and are mostly indirect. Conversely, AZA is generally considered safe for the heart, with no consistent association with arrhythmias in SLE studies so far [66].

DMARDs such as HCQ, AZA, MMF, CYC, and MTX are widely used in the management of rheumatic diseases. Our previous and extensive research on arrhythmogenesis across different autoimmune disorders has revealed a discrepancy in the volume of published evidence for SLE. The available literature indicates that, in rheumatoid arthritis, MTX may confer CV protection through its anti-inflammatory effects. Several studies have demonstrated an inverse association between MTX use and the prevalence of cardiac arrhythmias. Meanwhile, the studies present conflicting findings on HCQ in rheumatoid arthritis, indicating no association with an increased risk of arrhythmia [67].

In systemic sclerosis, evidence supporting a CV benefit of AZA remains inconclusive, with reports suggesting a potential risk of myocarditis or toxic myocardial injury. Regarding CYC, it may reduce inflammation, but high-dose therapy has been associated with significant cardiotoxicity, including AF, supraventricular tachycardia, premature ventricular contractions, ventricular tachycardia, high-grade atrioventricular block, cardiomyopathy, and heart failure. In both systemic sclerosis and SLE, the available evidence supporting the CV benefits of MMF remains insufficient. Reported cardiac rhythm disturbances are rare and include supraventricular tachycardia, atrial and ventricular ectopy, and bundle branch block [68].

The absence of robust, drug-specific data underscores a significant knowledge gap, and individualized cardiac monitoring remains essential for SLE patients receiving immunosuppressive therapy.

#### 3.5.2. bDMARDs

Systemic lupus erythematosus can affect multiple organs, especially the heart. Therefore, treatment depends on the type and severity of symptoms and the degree of organ involvement. For moderate-to-severe cases, therapy may be intensified with high-dose (pulse) corticosteroids and biologic agents such as belimumab or anifrolumab, which offer targeted disease management [69].

**Belimumab** is a recombinant human immunoglobulin G1λ monoclonal antibody that binds to and inhibits the soluble B-lymphocyte stimulator protein, thereby reducing B-cell survival. It has been approved in multiple countries for patients aged ≥5 years with active, autoantibody-positive SLE, as well as for adults with lupus nephritis receiving standard therapy. Numerous clinical trials have consistently demonstrated the safety and efficacy of belimumab. Furthermore, additional safety data from randomized trials in specific patient subgroups indicate that belimumab is generally well tolerated [70].

A real-world observational study following the STROBE guidelines analyzed adverse events related to Saphnelo and Benlysta using data from the FDA Adverse Event Reporting System. Data collected from April 2011 to April 2024 resulted in 21,631 cases, with 20,819 linked to belimumab. No cases of arrhythmia were reported after treatment, but information on cardiac adverse effects remains insufficient [71].

Cardiovascular disease, particularly atherosclerosis, is a significant complication in SLE. Belimumab has demonstrated effectiveness in reducing disease flares and may exert athero-protective effects. Nevertheless, a reported case describes a 40-year-old male with SLE and refractory lupus nephritis who, despite treatment with corticosteroids, MMF, and belimumab, developed recurrent palpitations and was diagnosed with non-calcified coronary atherosclerotic plaques. Notably, the palpitations recurred consistently after belimumab administration. Following the switch to telitacicept, the patient’s palpitations subsided, complement levels showed improvement, and proteinuria significantly decreased. This case highlights the potential role of telitacicept as a promising therapeutic option for refractory active SLE complicated by atherosclerosis, particularly in patients with suboptimal response or intolerance to belimumab [72].

**Anifrolumab** suppressed key inflammatory mediators associated with the IFN-I pathway, including NETs, TNF, and IL-10. In addition, it improved cardiometabolic markers, including glycoprotein acetylation and cholesterol efflux capacity, relative to baseline. Together, these results indicate that, by inhibiting the IFN-I pathway, anifrolumab may help mitigate CV risk in patients with SLE [73].

The evaluation of anifrolumab was conducted in three randomized, double-blind, placebo-controlled 52-week trials: MUSE (phase II) and TULIP-1 and TULIP-2 (phase III). Across studies, monthly intravenous anifrolumab 300 mg demonstrated superior efficacy versus placebo, improving composite disease activity scores, reducing glucocorticoid use, and lowering flare rates. Safety monitoring included a Data Safety Monitoring Board in all trials and a Cardiovascular Event Adjudication Committee in TULIP-1 and TULIP-2. Major adverse CV events were rare: one non-fatal myocardial infarction occurred in the anifrolumab group and none in the placebo group. In MUSE, no CV events were reported with anifrolumab, compared with three in the placebo group. Laboratory values, blood pressure, heart rate, and ECG findings remained comparable between groups, indicating a favorable CV safety profile and no evidence of arrhythmogenic or hemodynamic effects in SLE patients [74].

A series of FDA Adverse Event Reporting System reports from Q4 2021 to Q3 2024, in which anifrolumab was the primary suspected drug, was examined. The most frequently reported events included upper respiratory tract infection, bronchitis, infusion-related reactions, herpes zoster, cough, hypersensitivity, and nasopharyngitis. Additional potential reactions not listed on the product label included dyspnea, pyrexia, vomiting, pruritus, dizziness, chest pain, urticaria, alopecia, increased blood pressure, swelling, and migraine, most occurring within the first month of treatment. Data analysis identified rare reports of cardiac rhythm disturbances associated with anifrolumab. Specifically, two cases of atrial flutter were reported, corresponding to a reporting odds ratio of 8.85%, and two cases of supraventricular tachycardia with an odds ratio of 7.5%. These findings suggest that, while arrhythmias have been reported, they remain uncommon in patients receiving anifrolumab. There were no other positive signs of fatal adverse reactions [75].

#### 3.5.3. Additional Therapy

Glucocorticoids remain central to SLE treatment because of their potent anti-inflammatory effects, including reduced cytokine expression, inhibited leukocyte migration, and modulation of immune cell function. Up to 88% of SLE patients receive glucocorticoids, and over half continue long-term therapy. However, glucocorticoid treatment contributes significantly to both early and late organ damage, which is particularly concerning because irreversible damage predicts increased morbidity and mortality in SLE. Current guidelines recommend restricting or discontinuing glucocorticoids based on disease activity, treatment duration, and therapeutic response to minimize serious complications while maintaining disease control [76].

Corticosteroid therapy may cause microvascular occlusion in patients with pre-existing arterial endothelial damage. This occurs because corticosteroids reduce prostacyclin (which normally widens blood vessels and prevents platelet clumping) and increase thromboxane A2 (which narrows vessels and promotes platelet aggregation). As a result, increased platelet aggregation can lead to thrombosis and blockage of small arteries, such as nodal arteries, which may explain the conduction disturbances observed in these patients. The literature reports the case of an adult with SLE, positive for LAC, who developed a variety of arrhythmias and conduction disturbances shortly after starting corticosteroid therapy [77].

### 3.6. Screening and Management Strategies

Cardiovascular disease and subsequently cardiac arrhythmias in SLE is often under-recognized when assessment relies exclusively on traditional risk factors. QRISK3, a CV risk prediction tool, incorporates SLE and corticosteroid use as disease-specific variables, improving the detection of CVD risk in patients with SLE. Traditional calculators (SCORE or ASCVD) often underestimate CV risk in SLE populations because they primarily target older adults and do not account for the impact of chronic systemic inflammation, high disease activity, lupus nephritis, and long-term corticosteroid exposure. By identifying high-risk individuals earlier, QRISK3 supports proactive preventive strategies, including adapted pharmacologic interventions, lifestyle modifications, and optimization of immunosuppressive therapy. This approach facilitates personalized CV care and may reduce the incidence of major CV events in the SLE population [78].

Recent evidence indicates that early detection requires integrating laboratory markers with advanced CV imaging. Serological indices, including autoantibodies, disease-activity markers, history of lupus nephritis, exposure to immunosuppressive agents, and genetic predisposition, can identify individuals at increased CV risk even when standard predictors are unremarkable. Advanced imaging modalities, such as echocardiography, carotid ultrasound, coronary computed tomography for calcium scoring and plaque assessment, and particularly CV magnetic resonance imaging, can detect subclinical myocardial inflammation, early atherosclerosis, valvular abnormalities, and vascular wall changes in asymptomatic patients. The combination of laboratory indices with multimodal imaging enables a more precise assessment of early CV involvement, facilitating timely preventive interventions and improving long-term outcomes in patients with SLE [79].

In a comparative study of SLE patients with and without LSE, those with LSE had a higher prevalence of APAs and a lower prevalence of SLE-specific autoantibodies. Clinically, transthoracic echocardiography is recommended for initial screening in SLE patients with triple aPL positivity, and transesophageal echocardiography is used for confirmation [16].

Echocardiographic screening of asymptomatic SLE patients often reveals subclinical cardiac abnormalities, reflecting the silent burden of lupus-related CVD. Common findings include valvular thickening or regurgitation (particularly Libman–Sacks vegetations), pericardial effusion, diastolic dysfunction, and mild pulmonary hypertension. These abnormalities correlate with disease duration, inflammatory activity, and the presence of APAs. Routine echocardiography can therefore play a crucial role in early detection of CV involvement, enabling timely intervention and improved long-term outcomes in SLE [80]. A meta-analysis of 20 case–control studies, including 1117 patients with SLE and 901 healthy controls, found that cardiac involvement is common in SLE, even without clinically overt heart disease. Compared with controls, SLE patients had markedly higher odds of pericardial effusion (OR 30.52) and combined valvular abnormalities (OR 11.08), as well as increased left atrial diameter, left ventricular diastolic diameter, and left ventricular mass index. These findings support the routine use of echocardiography for early detection of subclinical cardiac involvement in SLE [81].

The 2019 EULAR recommendations highlight that patients with SLE should undergo routine evaluation for both traditional and disease-specific CV risk factors. These include persistent active disease, longer disease duration, medium-to-high titers of APAs, renal involvement, and chronic glucocorticoid therapy. Based on an individual’s overall CV risk profile, preventive strategies such as low-dose aspirin or lipid-lowering medications may be appropriate [82].

A cohort of 291 SLE patients without prior CV events was followed for a median of eight years to assess the effect of preventive therapy. During follow-up, 16 new CV events occurred. Multivariate analysis showed that regular low-dose aspirin use was associated with a 76% lower risk of a first CV event (HR≈ 0.24), and long-term HCQ (taken for more than five years) was associated with a 73% lower risk (HR ≈ 0.27). In contrast, no evident preventive effect was observed with statin therapy in this cohort. The authors concluded that aspirin and HCQ appear to offer additive benefits in primary prevention of CV events in SLE, though previous literature had shown conflicting results, likely due to shorter follow-up periods and study design limitations [83].

Additionally, current preconception and pregnancy management guidelines for women with SLE emphasize multidisciplinary care and optimal disease control, with recommendations primarily focused on preventing and managing fetal congenital heart block rather than maternal conduction disturbances. A published case report describes the incidental diagnosis of asymptomatic complete heart block during pregnancy in a woman with SLE and gestational hypertension treated with methyldopa, a drug with known potential to impair atrioventricular conduction. After a multidisciplinary risk–benefit evaluation, permanent pacemaker implantation was successfully performed during pregnancy to avert hemodynamic compromise and intrauterine growth restriction, with favorable outcomes for both mother and neonate. This case highlights a possible interplay between SLE, antihypertensive therapy, and maternal complete heart block, underscoring a notable gap in current clinical guidelines and the need for enhanced CV monitoring and individualized management in women with SLE of reproductive age [84].

**Figure 2:**
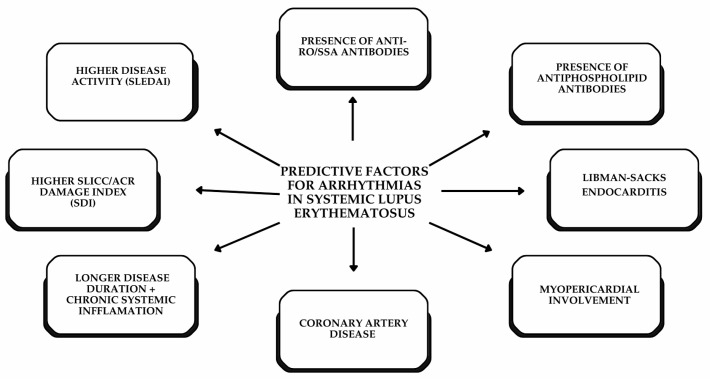
Predictive factors of arrhythmias in systemic lupus erythematosus.

Structured multidisciplinary care conduits that integrate rheumatology, obstetrics/gynecology, hematology, cardiology, and allied specialties improve the management of women with SLE and/or APS. These pathways call attention to preconception counseling, individualized risk stratification, optimization of disease control before pregnancy, standardized anticoagulation strategies for APS, and coordinated monitoring during pregnancy and the postpartum period. Published evaluations show that multidisciplinary models increase adherence to guideline-recommended therapies, reduce preventable complications (e.g., thromboembolism, severe flares, pregnancy loss), and improve patient counseling and care continuity. Consequently, multidisciplinary clinics are recommended as best practice for complex female SLE/APS patients, particularly those planning pregnancy, with high CV or thrombotic risk (Figure 2) [85].

## 4. Future Perspectives and Research Directions

Systemic Lupus Erythematosus is a complex autoimmune disease that affects many organs and shows a wide range of symptoms, with periods of flaring up and resolution. Managing the disease is challenging; current treatments are based on immunomodulatory and immunosuppressive drugs, depending on the organs involved, in order to achieve remission. The standard treatments, especially long-term use of glucocorticoids, can cause serious side effects and lead to lasting organ damage and higher irreversible health problems. Because of these issues, there is a growing shift toward a treat-to-target approach. This strategy focuses on basic therapies such as antimalarials, initiating steroid-sparing drugs or biologics early, minimizing glucocorticoid use, and closely monitoring other comorbidities. New targeted treatments that act on B-cells, interferon pathways, calcineurin inhibitors, and new small molecules may allow for more precise control of the immune system with fewer side effects. These advances are promising and could lead to better disease management [86].

Chimeric antigen receptor (CAR)-T cell therapy represents a major advancement in cellular immunotherapy. Dual-target CAR-T therapies simultaneously targeting CD19 on B-cells and the B-cell maturation antigen on plasma cells have demonstrated promising results. Early clinical data, including findings from Mackensen et al., reveal rapid and sustained B-cell depletion, improved complement levels, reductions in pathogenic autoantibodies, and durable remission in patients with refractory multi-organ SLE [87]. Subsequent studies validate high remission rates, reconstitution of a naïve B-cell compartment, and manageable safety profiles, with mostly mild-to-moderate cytokine release syndrome [88,89]. While the therapy has shown remarkable efficacy, CV considerations remain relevant in this patient population and necessitate appropriate monitoring [90]. Overall, dual-target CAR-T therapy represents a first-in-class, precision immunotherapy for refractory SLE, offering the potential for long-term disease control and immunologic reset. As clinical experience grows, prospective studies will be critical for confirming durability, optimizing treatment protocols, and evaluating systemic safety, including CV outcomes.

In conclusion, SLE causes cardiovascular involvement that is associated with arrhythmias through various and complexes mechanisms, mainly, related to direct cardiovascular structural damage, systemic inflammation or specific therapies. Data on arrhythmias secondary to cardiovascular damage in patients with SLE in the literature are limited. Improving outcomes depends on early detection, careful evaluation of therapy risks, and close collaboration between rheumatology and cardiology. A structured, multidisciplinary approach is essential to reduce morbidity, support individualized care, and enhance long-term CV prognosis for patients with SLE.

## Figures and Tables

**Table 1:** Summary of key data related to arrhythmias epidemiology in systemic lupus erythematosus (SLE).

Arrhythmia Type	Study Type, Population Studied and Follow-Up	Main Findings and Incidence/Prevalence, Risk Ratios/Hazard Ratios
**Supraventricular arrhythmias** Sinus tachycardia **Supraventricular tachycardia**/Atrial fibrillation	Narrative review summarizing multiple heterogeneous SLE cohorts from published studies, follow-up not applicable [39]. Holter monitoring study on 317 SLE patients (24-h Holter) [39]. Systematic review and meta-analysis of 6 cohort studies including 78,134 SLE patients vs. 347,883 controls; long-term follow-up [38]. Retrospective cohort study using an administrative database (2016–2019); 41,004 SLE hospitalizations [40].	Sinus tachycardia is the most common arrhythmia; in SLE; usually linked to systemic inflammation or disease flares; often transient and sometimes the sole cardiac manifestation. The predominant abnormalities observed were supraventricular ectopy (63.4%), atrial tachycardia (15.5%), and atrial fibrillation (2.8%). SLE is associated with increased AF risk (RR 1.85; 95% CI 1.23–2.79) and marks geographic variation (Europe/America vs. Asia). 3.65% had AF; AF was associated with higher in-hospital mortality (aOR 2.07), longer stay, and higher risk of MI, pericardial effusion, tamponade, cardiogenic shock.
**Ventricular arrhythmias**	Nationwide registry study of incident SLE patients without prior CVD; up to 10 years [27].	The 10-year absolute risk of ventricular arrhythmia, ICD implantation, or cardiac arrest was 0.89% (95% CI 0.58–1.31%)
**Conduction disease and brady-arrhythmias**	Nationwide registry study of incident SLE patients without prior CVD; up to 10 years [27]. Prospective cohort (Toronto Lupus Clinic); 1366 SLE patients, long-term follow-up [41].	Absolute risk of pacemaker implantation, AV block, or SA dysfunction: 0.59% (95% CI 0.36–0.94%). Rare but clinically significant over long-term follow-up. 18 (1.32%) required permanent pacemaker: 13 for complete AV block, 5 for sick sinus syndrome Brady-arrhythmias in SLE are mainly infrequent and mild. Severe conduction disorders requiring pacemaker implantation occur in about 1% of patients and are associated with traditional cardiovascular risk factors and prolonged antimalarial therapy exposure.

**Table 2:** Systemic Lupus Erythematosus Disease Activity Index.

Weight	Description	Definition
8	Seizure	Recent onset, exclude metabolic, infectious or drug causes
8	Psychosis	Altered ability to function in normal activities caused by severe disturbances in the perception of reality. Includes hallucinations, incoherence, marked mental association, impoverished thinking content, marked loss of logical thinking, or catatonia. Uremia and drug-induced symptoms should be excluded
8	Organic brain syndrome	Altered mental function with impaired orientation, memory or other intellectual functions, with rapid onset and fluctuations in the severity of clinical symptoms; includes eclipse of consciousness with decreased ability to focus attention and inability to maintain attention and at least two of the following symptoms: impaired perception, incoherent speech, insomnia or daytime sleepiness, increased or decreased psychomotor activity. Symptoms caused by metabolic disorders, infection or medication should be excluded
8	Visual disturbance	Retinal changes of SLE. These include cytoid bodies, retinal hemorrhage, serous or hemorrhagic exudate into the choroid, or optic neuritis. Hypertension, infection or drug effects should be excluded
8	Cranial nerve disorder	Recent onset of sensory or motor neuropathy involving the cranial nerves
8	Lupus headache	Severe, persistent headache, which can be migraines, but does not resolve after narcotic analgesia
8	Cerebrovascular stroke	Recent onset of cerebrovascular accidents. Atherosclerosis should be excluded
8	Vasculitis	Ulceration, gangrene, tender nodules on the fingers, periungual infarctions, splinter hemorrhage or vasculitis confirmed by biopsy or angiogram
4	Arthritis	≥2 joints—pain and features of inflammation (tenderness, swelling or effusion)
4	Myositis	Proximal muscle aching/weakness associated with elevated creatine kinase/aldolase activity or with electromyogram changes, or biopsy result indicating myositis
4	Urinary casts	Heme-granular or red blood cell casts
4	Hematuria	>5 erythrocytes in the field of view when evaluating urine sediment; exclude stones, infection and other causes
4	Proteinuria	>0.5 g/24 h
4	Pyuria	>5 leukocytes in the field of view, when evaluating urine sediment; exclude infection
2	Rash	Inflammatory type rash
2	Alopecia	Abnormal, patchy or diffuse loss of hair
2	Mucosal ulcers	Oral or nasal ulcerations
2	Pleuritis	Pleuritic chest pain with pleural rub or pleural effusion or pleural thickening
2	Pericarditis	Pain accompanied by at least one of the following: rubbing, effusion, confirmation of pericarditis on electrocardiogram or ultrasound
2	Low complement	Decreased levels of complement components C3, C4 or impaired CH50 hemolytic activity
2	Increased DNA binding	Increased DNA binding in the Farr test above the standard for laboratory tests
1	Fever	>38 °C; exclude infections
1	Thrombocytopenia	<100,000/mm^3^; exclude drug causes
1	Leukopenia	<3000/mm^3^; exclude drug causes

## Data Availability

During the preparation of this manuscript, the authors used ChatGPT 5.2 for the purpose of language correction. The authors have reviewed and edited the output and take full responsibility for the content of this publication.

## Abbreviations

The following abbreviations are used in this manuscript:

AF	Atrial Fibrillation
ANTI-Β2GPI	Anti-Β2-Glycoprotein-I Antibodies
APAa	Antiphospholipid antibodies
APS	Antiphospholipid Syndrome
AZA	Azathioprine
CAD	Coronary Artery Disease
CAR-T	Chimeric Antigen Receptor
CMR	Cardiac Magnetic Resonance
CSDMARDS	Conventional Synthetic Disease-Modifying Antirheumatic Drugs
CTDS	Connective Tissue Diseases
CV	Cardiovascular
CVD	Cardiovascular Disease
CYC	Cyclophosphamide
DMARDS	Disease-Modifying Antirheumatic Drugs
ECG	Electrocardiography
ESCAR	Echocardiography With Ultrasound Multi-Pulse Scheme
FQRS	Fragmented QRS
HCQ	Hydroxychloroquine
IL-1Β	Interleukin-1 Beta
IL-6	Interleukin-6
LAC	Lupus Anticoagulant
LSE	Libman-Sacks Endocarditis
MMF	Mycophenolate Mofetil
MTX	Methotrexate
SLE	Systemic Lupus Erythematosus
TNF-A	Tumor Necrosis Factor-Alpha
